# Multidimensional Structural Echocardiographic Patterns and Risk Score for Prognostic Stratification in Ischemic Cardiomyopathy

**DOI:** 10.3390/jcm15114386

**Published:** 2026-06-05

**Authors:** Ruixuan Tang, Yan Xu, Xiao Zong, Roubai Pan, Suyi Jia, Rui Xi, Rong Tao, Qin Fan

**Affiliations:** 1 Departments of Cardiology, Rui Jin Hospital, Shanghai Jiaotong University School of Medicine, 197 Rui Jin Road II, Shanghai 200025, China; hilda_tang@sjtu.edu.cn (R.T.); xy11374@rjh.com.cn (Y.X.); zx01m20@rjh.com.cn (X.Z.); prb01m16@rjh.com.cn (R.P.); jsyhappy0920@sjtu.edu.cn (S.J.); xr11420@rjh.com.cn (R.X.); 2 Institute of Cardiovascular Diseases, Shanghai Jiaotong University School of Medicine, Shanghai 200025, China

**Keywords:** ischemic cardiomyopathy, echocardiography, structural remodeling, ventricular dilation, pre-revascularization assessment

## Abstract

**Background**: Ischemic cardiomyopathy (ICM) is characterized by heterogeneous structural remodeling that is not fully captured by conventional systolic metrics. How multidimensional structural echocardiographic information can improve pre-revascularization risk stratification remains unclear. **Methods**: In this retrospective study, 989 patients with ICM undergoing coronary angiography and revascularization were included in the derivation cohort, and 482 patients from an independent campus served as the validation cohort, with a median follow-up duration of 6.5 years. The primary endpoint was cardiovascular mortality. Eight routinely acquired pre-revascularization echocardiographic structural variables were analyzed. Unsupervised clustering identified structural clusters, and principal component analysis (PCA) was used to derive a structural risk score. Associations with cardiovascular mortality were assessed using the Cox proportional hazards model, and prognostic performance was evaluated by comparing individual echocardiographic predictors using Harrell’s C-index and time-dependent AUC analyses. **Results**: Three distinct structural clusters emerged, differing in chamber size, systolic function, pulmonary pressures, mitral regurgitation severity, and long-term cardiovascular mortality. The PCA-derived structural risk score, reflecting the dominant axis of remodeling and volume overload, showed association with cardiovascular mortality in the derivation cohort and remained independently predictive after multivariable adjustment. Compared with single echocardiographic parameters, both the structural clusters and the risk score demonstrated superior discriminative performance. In the validation cohort, the structural score again showed a consistent and independent association with cardiovascular mortality. **Conclusions**: Multidimensional structural echocardiographic assessment reveals clinically meaningful remodeling patterns and enables construction of a robust PCA-derived structural risk score. Both approaches provide prognostic information beyond individual echocardiographic measures and support more precise pre-revascularization risk stratification in patients with ICM.

## 1. Introduction

Ischemic cardiomyopathy (ICM) is a major cause of heart failure and cardiovascular mortality, driven by chronic ischemia that leads to progressive left ventricular remodeling, chamber dilation, and systolic dysfunction [1,2,3]. Accurate pre-revascularization risk stratification is therefore crucial for guiding revascularization decisions and anticipating postoperative recovery [4]. Although left ventricular ejection fraction (LVEF) remains the most commonly used marker of systolic performance, it reflects only one aspect of ventricular function and often fails to capture the broader structural changes that shape long-term prognosis [5].

Echocardiography characterizes multiple dimensions of cardiac structure and loading conditions, including chamber size, wall thickness, pulmonary pressures, and valvular regurgitation [6]. These measures provide a more complete picture of ventricular remodeling and hemodynamic burden [7]. However, current risk assessment strategies rarely integrate this multidimensional information [8]. This gap leaves substantial heterogeneity in structural remodeling and limits the ability to achieve more precise pre-revascularization risk stratification. In ischemic cardiomyopathy, where chronic ischemia drives complex and heterogeneous patterns of ventricular remodeling, this limitation is particularly consequential. Although recent machine-learning studies have shown that high-dimensional echocardiographic features can be leveraged to improve risk prediction and phenotypic characterization, these approaches often rely on complex, non-interpretable models and are typically trained on broad, heterogeneous populations rather than disease-specific cohorts [9,10]. This underscores the need for clinically interpretable, structure-based methods capable of capturing remodeling heterogeneity in a scalable and disease-specific manner.

To better characterize the heterogeneity in pre-revascularization structural remodeling, we applied unsupervised clustering to eight routinely acquired echocardiographic variables in a large ICM cohort and identified three distinct structural clusters with markedly different prognostic profiles. We also derived a structural risk score using principal component analysis and evaluated its performance in an independent cohort. Both the stratification and the structural score independently predicted cardiovascular mortality and demonstrated superior discrimination compared with individual echocardiographic measures.

These findings suggest that multidimensional structural assessment may carry significant prognostic value and that integrating this information could enhance pre-revascularization risk stratification and help inform more individualized management in patients with ICM.

## 2. Methods

### 2.1. Patient Enrollment

This retrospective observational study was conducted at Shanghai Ruijin Hospital (the main and north campuses). We screened patients hospitalized between 2010 and 2018 with a diagnosis of ICM. All patients included had undergone coronary angiography and received revascularization procedures at our center. Transthoracic echocardiography was performed after admission and prior to revascularization in all patients. ICM was defined according to guideline criteria and confirmed independently by two cardiologists, requiring significant coronary stenosis on angiography (≥70% in a major epicardial vessel or ≥50% in the left main) together with left ventricular structural or functional abnormalities on echocardiography. Patients with non-ischemic cardiomyopathies, acute myocardial infarction at admission, or severely incomplete data were excluded, yielding a final derivation cohort of 989 patients. A validation cohort was assembled from the hospital’s north campus, a geographically distinct site within the same medical system, staffed by separate admitting teams, revascularization operators, and echocardiography personnel. This provided a naturally independent patient sample for out-of-sample validation. Using identical inclusion criteria and endpoint adjudication, 482 patients were included.

### 2.2. Echocardiographic Acquisition and Preprocessing

All echocardiographic studies were performed in accordance with the American Society of Echocardiography and European Association of Cardiovascular Imaging standards [7]. Eight structural variables were selected for analysis: left atrial diameter (LAd), left ventricular end-diastolic and end-systolic diameters (LVEDD and LVESD), LVEF, pulmonary artery pressure (PAP), mitral regurgitation (MR), interventricular septal thickness (IVS), and posterior wall thickness (PWT). PAP was defined as echocardiographically estimated systolic pulmonary artery pressure, calculated from the peak tricuspid regurgitant velocity using the modified Bernoulli equation and adding an estimate of right atrial pressure based on inferior vena cava size and collapsibility, in accordance with ASE/EACVI guidelines. MR was recoded from its original qualitative grades into an ordered 1–5 scale to retain ordinal information for multivariable analyses. These eight parameters were selected because they were consistently measured across the study period with near-universal availability. Advanced functional indices such as global longitudinal strain, E/e’, deceleration time, left atrial strain, and right ventricular strain were not incorporated due to inconsistent acquisition and substantial missingness, which would have reduced the effective sample size and affected the stability of unsupervised clustering.

### 2.3. Clustering Analysis and Structural Risk Score

Unsupervised clustering was performed in the derivation cohort using the eight variables. Partitioning Around Medoids (PAM) was selected because of its robustness to outliers and suitability for mixed-scale data. The optimal number of clusters was determined as k = 3 based on internal validation metrics together with clinical interpretability. Detailed comparisons of k = 2–4 solutions are provided in Supplementary Figure S1. To characterize structural variation along a continuous axis, principal component analysis (PCA) was applied to the same variables after z-score standardization, and the mean and standard deviation used for standardization in the derivation cohort are provided in Supplementary Table S1. Component stability was assessed through bootstrap resampling. The first principal component, which captured the dominant pattern of cardiac remodeling and volume overload, was used to construct a linear structural risk score by weighting each variable according to its loading. For univariable comparisons with individual echocardiographic measures, variables were additionally categorized into three levels using established clinical thresholds to allow direct comparison with the cluster-based groups, whereas comparisons involving the structural score used the original continuous variables.

### 2.4. Clinical Data Collection and Endpoint Adjudication

Demographic characteristics, comorbidities, laboratory results, and medication use were obtained from the electronic medical record system and anonymized before analysis. Data extraction was performed independently by two investigators. Missing clinical and laboratory data were handled using Multiple Imputation by Chained Equations (MICE) [11]. A detailed overview of missingness and imputation methods is presented in Supplementary Table S2. Follow-up information was collected through telephone interviews and review of electronic records, with a median follow-up duration of 6.5 years. The primary endpoint was cardiovascular death, defined according to ICD-10 codes I00-I99 or documented clinical evidence of a fatal cardiovascular event, including death due to myocardial infarction, heart failure, sudden cardiac death, stroke, fatal arrhythmia, or other vascular causes. Non-cardiovascular deaths were censored at the time of occurrence. All deaths were adjudicated by two independent cardiologists who reviewed hospital records, discharge summaries, imaging reports, death certificates, and follow-up information obtained from telephone interviews. In cases of disagreement, a third senior cardiologist provided the final determination. Adjudicators were blinded to clustering assignments, remodeling scores, and all model outputs. In total, 251 adjudicated cardiovascular deaths occurred in the derivation cohort (n = 989), and 116 in the validation cohort (n = 482).

### 2.5. Statistical Analysis

Continuous variables were summarized as mean ± SD or median (IQR), depending on distribution, and categorical variables as counts and percentages. Group differences were assessed using ANOVA or Kruskal–Wallis tests for continuous variables and chi-square or Fisher’s exact tests for categorical variables. Survival analyses were conducted with Cox proportional hazards models to estimate hazard ratios and 95% confidence intervals. The proportional hazards assumption was evaluated using Schoenfeld residuals for the echocardiographic clustering-based risk stratification and the structural risk score, and no violations were detected. For categorical cluster analyses, the low-risk cluster was consistently used as the reference group, so hazard ratios for the medium- and high-risk clusters were reported relative to the low-risk baseline. Model discrimination was quantified using Harrell’s C-index. For comparisons between structural risk stratification and individual echocardiographic predictors, time-dependent AUCs at 1, 3, and 5 years were computed using the timeROC approach. Incremental prognostic value was evaluated using changes in Harrell’s C-index (ΔC-index) and category-free net reclassification improvement (NRI). Decision curve analysis (DCA) was performed to assess the clinical utility of the structural risk score across a range of threshold probabilities. All statistical analyses were two-sided and conducted in R, using version 4.5.2 or 4.4.2 as appropriate based on package compatibility.

### 2.6. Ethics and Data Governance

The study protocol was approved by the Ethics Committee of Shanghai Ruijin Hospital (Approval No. 2016-019) and conducted in accordance with the Declaration of Helsinki.

## 3. Results

### 3.1. Pre-Revascularization Echocardiographic Clustering and Risk Stratification

**Figure 1:**
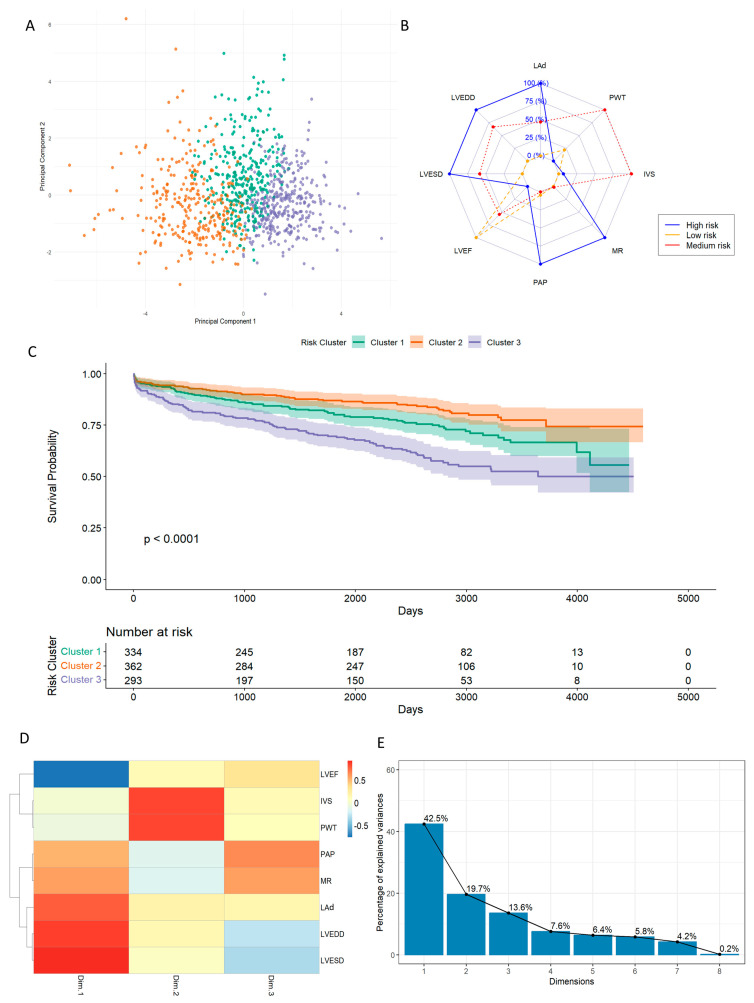
PCA-Derived Structural Patterns and Their Echocardiographic and Prognostic Profiles. (**A**) PCA projection showing consistent clustering structure in the principal component space. (**B**) Radar plot summarizing the multidimensional echocardiographic profiles of the three structural clusters. (**C**) Kaplan–Meier curves illustrating distinct long-term cardiovascular mortality trajectories across the three clusters. (**D**) Heatmap of PCA loadings. PC1 reflects the dominant axis of cardiac remodeling and volume overload, driven by chamber size, systolic function, pulmonary pressures, and mitral regurgitation; PC2 captures ventricular wall thickness characteristics; PC3 explains a smaller proportion of variance. (**E**) Percentage of variance explained by each principal component in the echocardiographic PCA. Abbreviations: LAd, left atrial diameter; LVEDD, left ventricular end-diastolic diameter; LVESD, left ventricular end-systolic diameter; LVEF, left ventricular ejection fraction; PAP, pulmonary artery pressure; MR, mitral regurgitation; IVS, interventricular septal thickness; PWT, posterior wall thickness.

Among the 989 patients included in the analysis, three distinct structural echocardiographic clusters emerged. The clusters showed clear separation in both PCA (Figure 1A) and Uniform Manifold Approximation and Projection (UMAP) spaces (Supplementary Figure S2A), supporting the internal consistency of the clustering structure. Clusters differed substantially in cardiovascular mortality, and based on their structural features and survival profiles, were designated as low-, medium-, and high-structural-risk groups (Figure 1B,C).

### 3.2. Baseline Characteristics Across Echocardiography-Derived Structural Risk Groups

To contextualize these structural clinical profiles, baseline characteristics were compared across groups (Table 1), with the high-risk group exhibiting a greater comorbidity burden and more adverse metabolic features. A full version of this table, including clinical, laboratory, and medication variables, is provided in Supplementary Table S3. Differences in cardiac structure and function were the most pronounced, with larger LAd, LVEDD, and LVESD, lower LVEF, higher PAP, and more severe MR in the high-risk group—patterns consistent with the PCA axis of remodeling and volume overload.

### 3.3. PCA Component Structure and Stability Analysis

To further characterize the structural patterns underlying the echocardiography-derived risk groups, we examined the component structure of the PCA model (Figure 1D,E, Supplementary Table S4). The first three principal components explained 42.5%, 19.7%, and 13.6% of the total variance (Figure 1E), respectively, indicating that PC1 captured the dominant source of variation among the eight structural variables. PC1 showed consistently high loadings on chamber size and loading parameters, including LAd, LVEDD, LVESD, PAP, and MR, with an inverse loading for LVEF, forming a coherent latent dimension representing global structural remodeling and hemodynamic burden. Therefore, it was selected as the basis for constructing the structural score. PC2 was driven primarily by ventricular wall thickness, with strong contributions from IVS and PWT. Although this component represented a distinct hypertrophic dimension, it did not align with the broader remodeling axis captured by PC1 and accounted for substantially less shared variance across the structural variables. Bootstrap resampling confirmed the stability of the loading structure, supporting the robustness of the PCA model (Supplementary Figure S2B).

### 3.4. Prognostic Performance of the Pre-Revascularization Structural Risk Stratification

**Figure 2:**
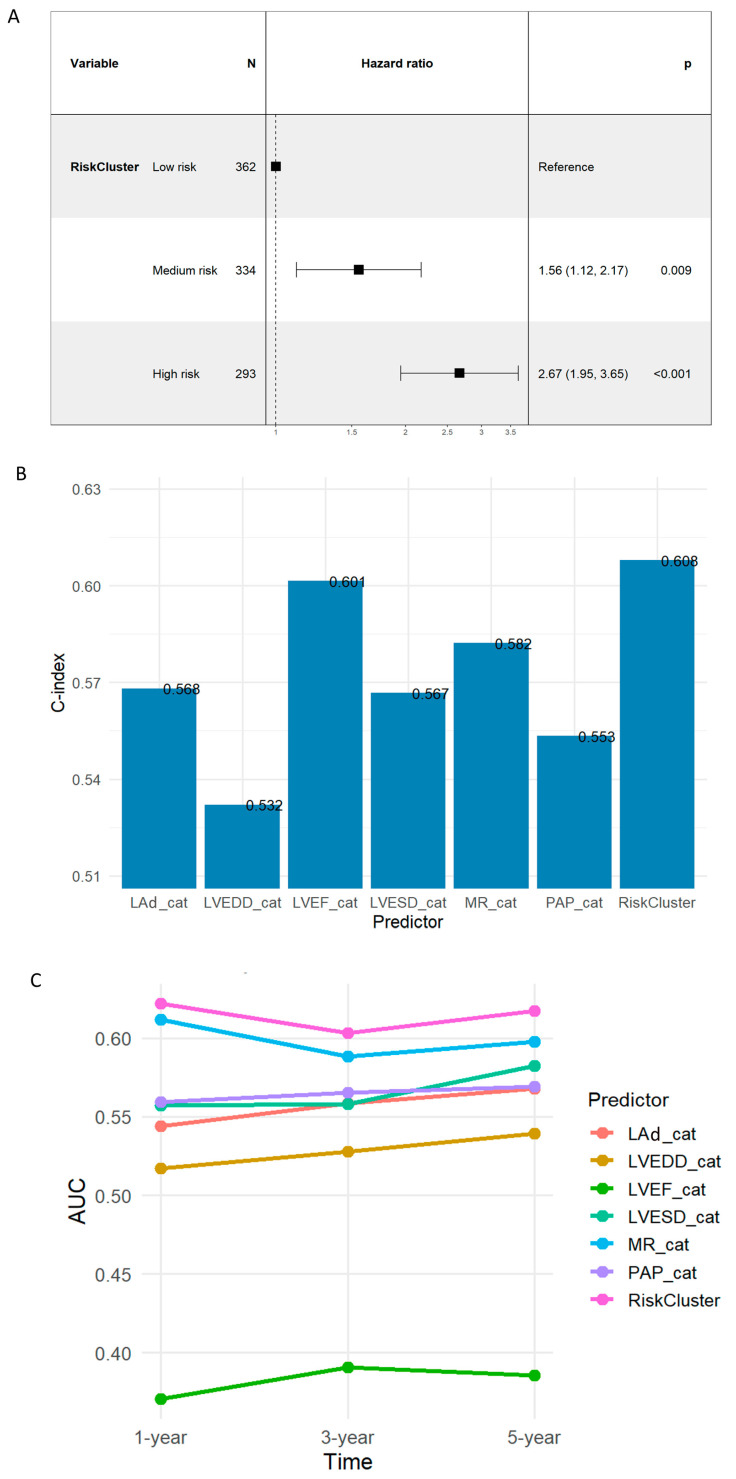
Discrimination performance of the structural risk cluster for cardiovascular mortality. (**A**) Unadjusted Cox proportional hazards model evaluating the association between pre-revascularization structural risk clusters and cardiovascular mortality. Hazard ratios (HRs) and 95% confidence intervals (CIs) are shown. (**B**) Harrell’s C-index for categorized echocardiographic predictors. (**C**) Time-dependent AUC trajectories for categorized echocardiographic predictors at 1, 3, and 5 years. Abbreviations: HR, hazard ratio; PAP, pulmonary artery systolic pressure; LAd, left atrial diameter; LVEDD, left ventricular end-diastolic diameter; LVESD, left ventricular end-systolic diameter; LVEF, left ventricular ejection fraction; MR, mitral regurgitation. Categorical variables (e.g., LAd_cat, LVEDD_cat, LVESD_cat, LVEF_cat, PAP_cat, MR_cat) represent clinically categorized versions of the corresponding echocardiographic parameters based on established threshold values. RiskCluster refers to the structural risk stratification.

Across all Cox regression models, the echocardiography-derived structural risk groups demonstrated a clear stepwise increase in cardiovascular mortality (Figure 2A, Supplementary Figure S3A–C). Adjustment for age and sex further confirmed these associations. In the adjusted model, which additionally included revascularization strategy, chronic kidney disease, NYHA class, and LDL cholesterol, the structural risk groups remained independently predictive. In an extended sensitivity model additionally adjusting for sex, diabetes, arrhythmias, systolic blood pressure, resting heart rate, and major cardiovascular medications (ACEI/ARB, beta-blockers and statins), the associations between the pre-revascularization structural risk groups and cardiovascular mortality remained robust, with effect sizes similar to those observed in the primary model. These results indicate that the prognostic value of the structural clusters was not driven by residual confounding from clinical comorbidities or treatment patterns.

### 3.5. Comparison Between Categorized Structural Clusters and Categorized Single Echocardiographic Predictors

To ensure a consistent comparison framework, all individual echocardiographic variables were evaluated in their categorized forms using established clinical thresholds. Compared with these single-parameter predictors, the structural clusters showed markedly superior prognostic discrimination, achieving the highest overall C-index and consistently outperforming all individual measures across 1-, 3-, and 5-year time-dependent AUCs (Figure 2B,C).

### 3.6. Prognostic Performance of the Structural Risk Score

Based on the loadings of the first principal component, a structural risk score was constructed as follows:

Structural score = 0.78⋅LAd + 0.87⋅LVEDD + 0.93⋅LVESD − 0.76⋅LVEF + 0.51⋅PAP + 0.58⋅MR

**Figure 3:**
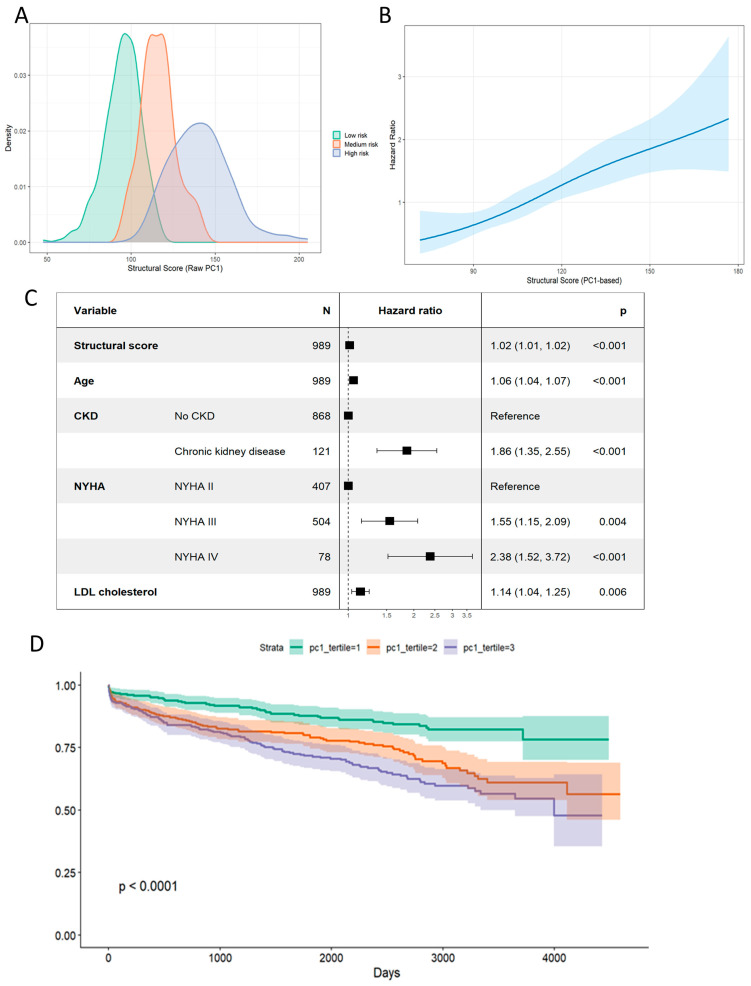
Prognostic validation of the structural risk score. (**A**) Density distributions of the continuous structural risk score across the three structural clusters. (**B**) Restricted cubic spline analysis showing a monotonic increase in cardiovascular mortality risk with higher structural risk scores. (**C**) Multivariable Cox regression analysis evaluating the prognostic value of the structural risk score. (**D**) Kaplan–Meier curves based on tertiles of the structural risk score.

**Figure 4:**
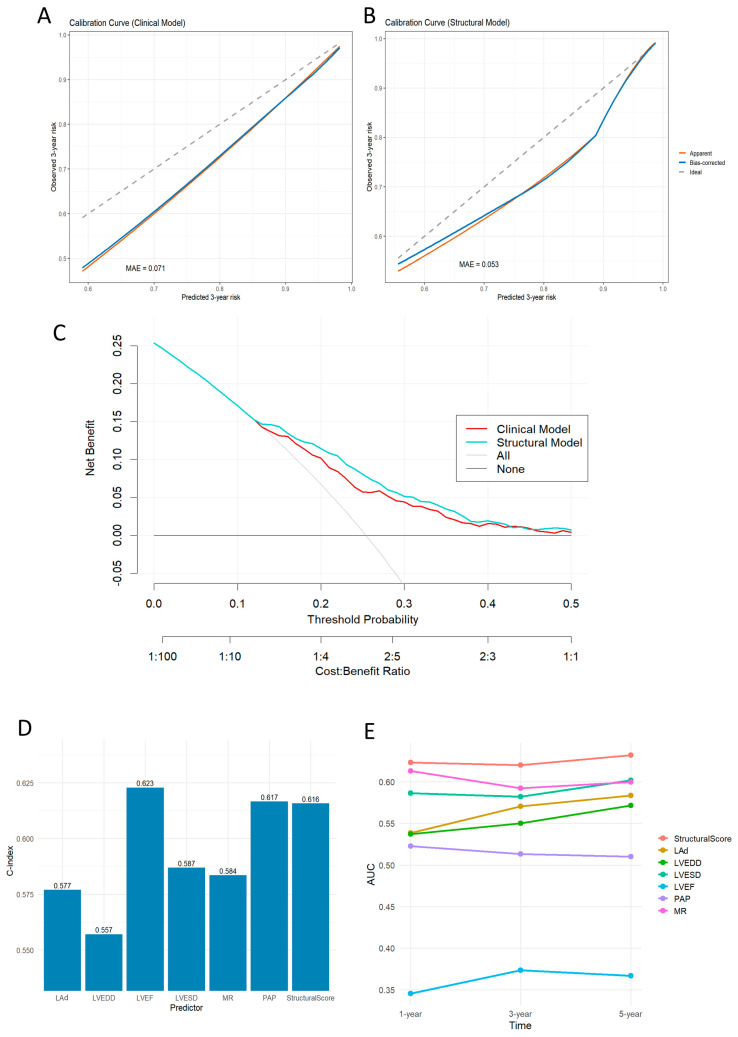
Comparative calibration, clinical utility, and discrimination performance of the structural score. The Clinical Model and Structural Model were constructed using Cox proportional hazards regression. The Clinical Model included Age, CKD, NYHA class, and LDL cholesterol. The Structural Model incorporated the structural risk score in addition to these same clinical covariates. (**A**) Calibration curve of the Clinical Model. (**B**) Calibration curve of the Structural Model. (**C**) Decision curve analysis (DCA) comparing the net clinical benefit of the Structural Model and Clinical Model. (**D**) Harrell’s C-index for individual echocardiographic and clinical predictors. (**E**) Time-dependent AUC for individual predictors at 1, 3, and 5 years. Abbreviations: PAP, pulmonary artery systolic pressure; LAd, left atrial diameter; LVEDD, left ventricular end-diastolic diameter; LVESD, left ventricular end-systolic diameter; LVEF, left ventricular ejection fraction; MR, mitral regurgitation; AUC, area under the curve; MAE, mean absolute error; DCA, decision curve analysis.

The score showed a clear separation across the three structural groups (Figure 3A). In the derivation cohort (n = 989), the score demonstrated a clear and graded association with cardiovascular mortality (Figure 3B–D). This association remained consistent across multiple model specifications, including an extended sensitivity model incorporating comorbidities, hemodynamic variables, and cardiovascular medications (Supplementary Figure S4). Beyond its independent prognostic association, the structural risk score meaningfully improved model performance when added to a clinical model containing age, CKD, NYHA class, and LDL cholesterol. Discrimination increased from a C-index of 0.687 to 0.714 (ΔC-index = 0.027; 95% CI 0.013–0.046). Risk reclassification at 3 years also improved substantially. The category-free NRI was 0.334, reflecting meaningful improvements in both event and non-event reclassification. Among patients who experienced events, slightly more than half were reassigned to a higher predicted risk, yielding a modest positive event-specific contribution (NRI+ = 0.036). Among those who remained event-free, nearly two-thirds were correctly reassigned to a lower-risk category, producing a large favorable non-event component (NRI− = 0.298). Overall, these patterns indicate that the structural score enhances upward reclassification for true events while substantially reducing overestimation of risk among non-events. Calibration analyses showed good agreement between predicted and observed 3-year risks, with the structural model demonstrating slightly better calibration than the clinical model (Figure 4A,B). Decision curve analysis further indicated that the structural model provided higher net benefit across clinically relevant threshold probabilities, supporting its greater utility for risk-based decision-making (Figure 4C).

### 3.7. Comparison Between the Structural Score and Individual Echocardiographic Predictors

To provide a consistent comparison framework, the prognostic performance of the continuous structural risk score was evaluated against each individual echocardiographic parameter in its original continuous form. Across both concordance statistics and time-dependent ROC analyses, the structural risk score ranked among the strongest predictors and demonstrated the most consistent overall performance (Figure 4D,E). Although LVEF and pulmonary artery pressure showed slightly higher C-indices, their time-dependent AUCs were inferior or modest, whereas the structural risk score remained high and stable.

### 3.8. Independent Validation of the Structural Risk Score

**Figure 5:**
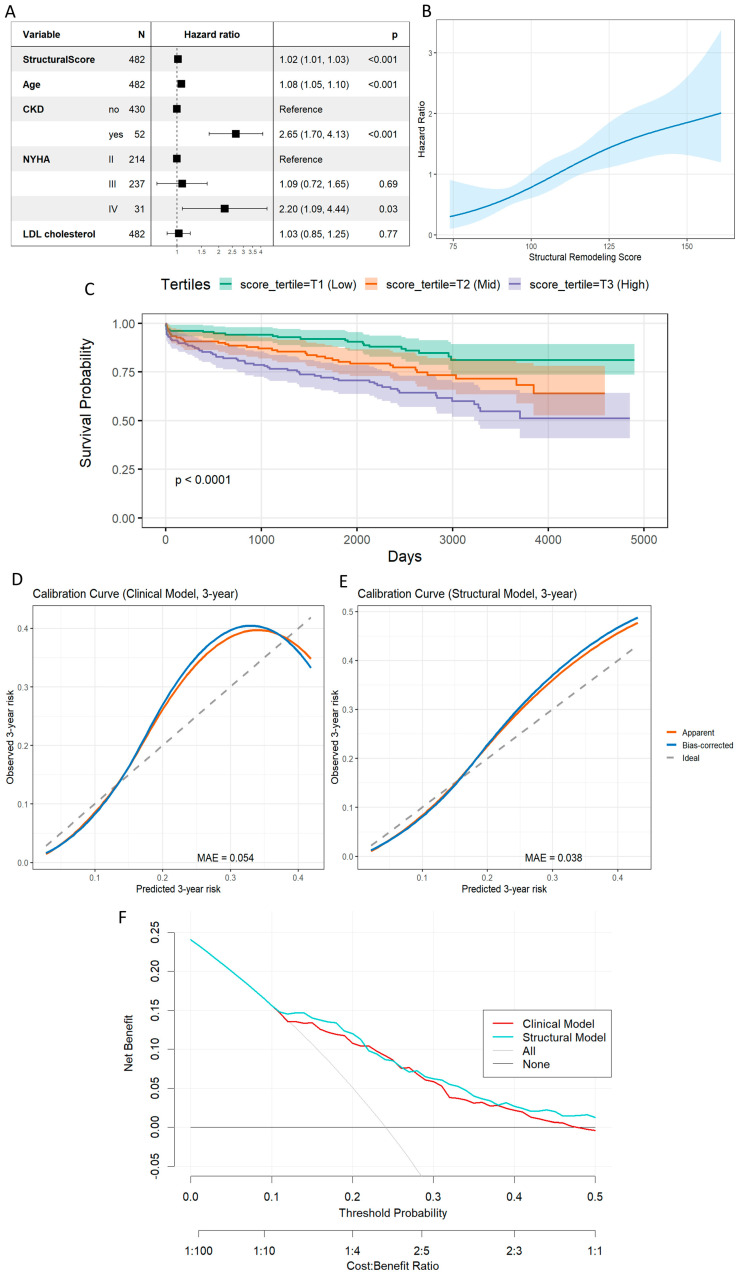
Validation of the Structural Risk Score. (**A**) Multivariable Cox regression analysis evaluating the prognostic value of the structural risk score in the validation cohort. (**B**) Restricted cubic spline analysis demonstrating a monotonic increase in cardiovascular mortality risk with higher structural risk scores. (**C**) Kaplan–Meier curves based on tertiles of the structural risk score. (**D**) Calibration curve of the Clinical Model. The Clinical Model included age, chronic kidney disease, NYHA class, and LDL cholesterol. (**E**) Calibration curve of the Structural Model. The Structural Model incorporated the structural risk score in addition to these same clinical covariates. (**F**) Decision curve analysis (DCA) comparing the net clinical benefit of the Structural Model and Clinical Model. Abbreviations: CKD, chronic kidney disease; NYHA, New York Heart Association functional class; LDL, low-density lipoprotein cholesterol; DCA, decision curve analysis.

In the validation cohort (n = 482), the structural risk score demonstrated strong reproducibility of its prognostic performance (Figure 5A–C). The score remained independently associated with cardiovascular mortality after adjustment for age, CKD, NYHA class, and LDL cholesterol, confirming that its predictive value was consistent across populations. When added to the same clinical model, the structural risk score improved discrimination, increasing the C-index from 0.750 to 0.774 (ΔC-index = 0.025; 95% CI 0.005–0.051), mirroring the improvement observed in the primary cohort. Risk reclassification at 3 years also showed a similar pattern of improvement. The category-free NRI was 0.353, driven by modest gains in upward reclassification among events (NRI+ = 0.069) and a larger improvement in correctly down-classifying non-events (NRI− = 0.284). Overall, 53.4% of cases were assigned higher predicted risk, and 64.2% of non-events were assigned lower predicted risk with the addition of the structural risk score, indicating better identification of true events and reduced overestimation of risk among non-events. Calibration analyses demonstrated good agreement between predicted and observed 3-year risks, with the structural model showing lower calibration error than the clinical model (Figure 5D,E). Decision curve analysis further showed higher net benefit across clinically relevant threshold probabilities, supporting the robustness and clinical utility of the structural risk score in a distinct patient population (Figure 5F).

## 4. Discussion

In this cohort of patients with ischemic cardiomyopathy, multidimensional pre-revascularization echocardiographic data revealed substantial heterogeneity in structural remodeling. Using eight routinely acquired parameters, unsupervised clustering identified three distinct structural patterns that differed in ventricular geometry, hemodynamic load, and long-term cardiovascular mortality. Based on these findings, we developed a structural risk score derived from the principal remodeling axis, which demonstrated consistent and independent prognostic value in both the derivation and validation cohorts. Together, these results highlight the importance of a multidimensional structural perspective beyond conventional systolic metrics. 

Traditional risk stratification in heart failure and ischemic cardiomyopathy has relied heavily on LVEF, yet LVEF captures only one dimension of ventricular function and often fails to reflect the broader structural and hemodynamic abnormalities associated with chronic ischemia [5]. Prior studies have shown that left atrial enlargement, ventricular dilation, elevated filling pressures, and functional mitral regurgitation each carry prognostic significance [12,13,14,15]. In our study, these interrelated markers aligned along a dominant structural axis associated with mortality and integrating them yielded substantially better prognostic discrimination than individual echocardiographic measures. Contemporary imaging frameworks underscore the fact that integrating structural and functional echocardiographic markers, early indicators of ventricular dysfunction such as strain, and CT-based coronary assessment substantially improves cardiovascular risk prediction. The anticipated incorporation of AI into these workflows further strengthens the rationale for multidimensional structural profiling [16].

We selected PAM clustering as the primary method because it provides stable partitions by using medoids, is less influenced by extreme values, and has been widely adopted in clinical phenotyping frameworks. As a robustness check, we also explored hierarchical clustering using Ward linkage. Although hierarchical clustering did not achieve the same level of separation as PAM, it nonetheless yielded broadly similar patterns, with cluster 2 remaining highly stable across methods (Supplementary Figure S5, Supplementary Table S5). This indicates that while PAM offered superior interpretability and stability for our dataset, alternative algorithms can still capture comparable structural groupings.

The three structural clusters identified in this study reflect distinct patterns of cardiac remodeling. The high-risk group showed pronounced chamber enlargement, impaired systolic function, elevated pulmonary pressures, and more severe mitral regurgitation, reflecting advanced adverse remodeling, and had the highest cardiovascular mortality. The association with cardiovascular mortality remained significant after adjustment for demographic and clinical factors, underscoring the independent prognostic value of structural echocardiographic profiles. Similar clustering approaches in heart failure populations have identified biologically coherent subgroups with differing outcomes [17,18,19]. Our findings extend this concept to ischemic cardiomyopathy, highlighting echocardiographic structural patterns.

Clinical and laboratory characteristics also differed consistently across clusters, suggesting that systemic disease burden and cardiac remodeling are closely linked. Patients in the high-risk group had more frequent arrhythmias, required more intensive diuretic therapy, and exhibited less favorable metabolic, renal, and neurohormonal profiles. These patterns indicate that structural remodeling may help contextualize clinical and biochemical markers within a broader disease framework. Although clusters differed in clinical and laboratory characteristics, sensitivity analyses additionally adjusting for a broad set of clinical covariates yielded similar hazard ratios for the associations between structural phenotypes and cardiovascular mortality, confirming that the prognostic value of the clustering remained robust despite these group-level differences.

Because sex distribution differed across structural groups, we performed sensitivity analyses adjusting for sex in both the continuous score and cluster-based models. Sex was not independently associated with cardiovascular mortality once the burden of structural remodeling and age were accounted for, whereas the structural score and cluster assignments remained strongly predictive. These findings suggest that sex imbalance did not materially influence the structural patterns or their prognostic performance. Nonetheless, prior studies have shown that men and women may follow distinct ventricular remodeling trajectories, including differences in chamber dilation, hypertrophy, and structural adaptation [20,21], indicating that sex-specific remodeling warrants further investigation in larger, prospectively enrolled cohorts with balanced sex representation.

The structural risk score provides a complementary, continuous measure of remodeling severity. Its consistent performance supports its generalizability and suggests potential clinical utility for risk stratification and for informing hypotheses regarding postoperative surveillance or follow-up intensity. Because the score relies on routinely acquired echocardiographic parameters, it is practical, scalable, and potentially implementable pending workflow integration and prospective validation. Future studies should benchmark the structural score against established risk scores to further define its comparative utility and potential role in clinical decision-making.

Several factors likely account for the stronger performance of the structural risk stratification compared with individual echocardiographic measures. In this cohort, most patients presented with markedly reduced systolic function, resulting in a compressed LVEF range that limited its ability to differentiate risk. In contrast, chamber size, pulmonary pressure, and mitral regurgitation varied more widely and contributed more information for risk separation. The PCA-based weighting further captured shared variance among correlated structural markers and reduced measurement noise, with LVESD, LVEDD, and LAd carrying the largest coefficients. These features collectively enhanced discriminative performance.

This study has several strengths, including a large sample size, long-term follow-up, comprehensive structural echocardiographic assessment, unsupervised clustering, and validation in an independent cohort. Nonetheless, several limitations warrant consideration. The retrospective, single-center design and reliance on telephone follow-up for some cases may introduce misclassification bias. Advanced echocardiographic measures such as global longitudinal strain, diastolic function indices (e.g., E/e’ and deceleration time), and left atrial or right ventricular strain were not included. These functional parameters provide important prognostic and mechanistic insights, capturing myocardial deformation, diastolic filling pressures, and atrial–ventricular interaction that extend beyond structural remodeling alone [7,22,23,24]. However, their acquisition was inconsistent across the study period, with a substantial lack of data and dependence on vendor-specific software. Incorporating these measures would have reduced the effective sample size and compromised clustering stability. In addition, although we performed validation in a geographically distinct campus within the same medical system, this approach constitutes an internal–external validation rather than a fully independent external validation. Also, the identified clusters may partly reflect progressive remodeling severity rather than distinct mechanistic phenotypes, and broader validation integrating biomarkers, coronary anatomy, and advanced imaging will be required to delineate mechanistic heterogeneity.

Future research should integrate structural profiling with biomarkers, coronary anatomy, and advanced imaging to capture both structural and biological determinants of risk. Prospective studies are needed to evaluate whether structural patterns can guide follow-up intensity, therapeutic adjustment, and clinical decision-making. A more integrated approach may ultimately improve risk assessment and long-term outcomes in patients with ischemic cardiomyopathy.

## Figures and Tables

**Table 1:** Baseline Characteristics by Pre-Revascularization Risk Group.

Variable	Low	Medium	High	*p*
Age	67.32 ± 11.15	66.24 ± 10.03	64.98 ± 10.10	0.023
Sex				<0.001
Female	94 (26.0%)	35 (10.5%)	43 (14.7%)	
Male	268 (74.0%)	299 (89.5%)	250 (85.3%)	
BMI	23.72 ± 3.34	25.15 ± 8.08	23.90 ± 3.42	<0.001
Hypertension				<0.001
No	125 (34.5%)	79 (23.7%)	126 (43.0%)	
Yes	237 (65.5%)	255 (76.3%)	167 (57.0%)	
Diabetes				0.111
No	220 (60.8%)	180 (53.9%)	158 (53.9%)	
Yes	142 (39.2%)	154 (46.1%)	135 (46.1%)	
Dyslipidemia				0.002
No	239 (66.0%)	226 (67.7%)	229 (78.2%)	
Yes	123 (34.0%)	108 (32.3%)	64 (21.8%)	
CKD				0.004
No	334 (92.3%)	283 (84.7%)	251 (85.7%)	
Yes	28 (7.7%)	51 (15.3%)	42 (14.3%)	
COPD				0.565
No	333 (92.0%)	300 (89.8%)	264 (90.1%)	
Yes	29 (8.0%)	34 (10.2%)	29 (9.9%)	
AF				0.101
No	324 (89.5%)	298 (89.2%)	245 (83.6%)	
Paroxysmal	26 (7.2%)	19 (5.7%)	32 (10.9%)	
Permanent	0 (0.0%)	2 (0.6%)	1 (0.3%)	
Persistent	12 (3.3%)	15 (4.5%)	15 (5.1%)	
NYHA				<0.001
II	194 (53.6%)	143 (42.8%)	70 (23.9%)	
III	148 (40.9%)	172 (51.5%)	184 (62.8%)	
IV	20 (5.5%)	19 (5.7%)	39 (13.3%)	
LAd	39.81 ± 3.93	43.63 ± 3.93	47.96 ± 4.90	<0.001
LVEDD	52.24 ± 3.85	60.34 ± 3.90	64.35 ± 6.46	<0.001
LVESD	38.72 ± 3.47	46.73 ± 3.77	52.40 ± 6.63	<0.001
LVEF	49.94 ± 5.53	44.10 ± 5.92	37.07 ± 7.10	<0.001
PAP	37.82 ± 8.42	37.16 ± 8.42	49.79 ± 12.30	<0.001
MR	2.19 ± 0.66	2.20 ± 0.50	3.55 ± 0.95	<0.001
IVS	8.92 ± 1.14	9.99 ± 1.56	8.99 ± 1.37	<0.001
PWT	8.70 ± 0.85	9.13 ± 1.24	8.58 ± 1.16	<0.001

This table summarizes key demographic, clinical, and echocardiographic characteristics across the three pre-revascularization structural risk groups. Continuous variables are presented as mean ± SD and were compared across groups using ANOVA or Kruskal–Wallis tests as appropriate. Categorical variables were compared using Chi-square or Fisher’s exact tests. Abbreviations: BMI, body mass index; CKD, chronic kidney disease; COPD, chronic obstructive pulmonary disease; AF, atrial fibrillation; NYHA, New York Heart Association functional class; LAd, left atrial diameter; LVEDD, left ventricular end-diastolic diameter; LVESD, left ventricular end-systolic diameter; LVEF, left ventricular ejection fraction; PAP, pulmonary artery pressure; MR, mitral regurgitation; IVS, interventricular septal thickness; PWT, posterior wall thickness.

## Data Availability

Due to institutional and patient privacy restrictions, the datasets generated and analyzed during the current study are not publicly available. Data may be obtained from the corresponding author upon reasonable request and with appropriate institutional approvals.

## Supplementary Materials

The following supporting information can be downloaded at https://www.mdpi.com/article/10.3390/jcm15114386/s1. Figure S1: Internal validation metrics for clustering solutions with k = 2, 3 and 4. (A–C). Silhouette width distributions for the three clustering configurations. The k = 2 solution showed the strongest separation but formed an overly coarse two-cluster structure. The k = 3 solution achieved a balanced level of cohesion and separation with clearer clinical interpretability. The k = 4 solution exhibited reduced silhouette width, indicating over-partitioning and weaker cluster separation. (D) Gap statistic across k = 2–4. The gap statistic increased only minimally with higher k, suggesting limited additional structural information beyond three clusters. Confidence intervals reflect the bootstrap-based standard error estimated from 100 resampled reference datasets. (E) Dunn index for k = 2–4; Figure S2: Internal validation of the structural clustering framework. (A) UMAP demonstrating separation of the three structural clusters. (B) Bootstrap stability analysis showing the frequency distribution of loading correlations across 100 resamples, demonstrating the high reproducibility of the PCA component structure; Figure S3: Forest plots of Cox proportional hazards models evaluating the association between pre-revascularization structural risk clusters and cardiovascular mortality. Hazard ratios (HRs) and 95% confidence intervals (CIs) are shown for each covariate included in the models. (A) A model adjusted for age and sex. (B) The multivariable model adjusted for revascularization strategy, age, chronic kidney disease, NYHA class, and LDL cholesterol. (C) An extended sensitivity model additionally adjusted for diabetes, arrhythmia, systolic blood pressure, resting heart rate, and major cardiovascular medications. Abbreviations: ACEI, angiotensin-converting enzyme inhibitor; ARB, angiotensin receptor blocker; LDL, low-density lipoprotein cholesterol; BP, blood pressure; CKD, chronic kidney disease; NYHA, New York Heart Association functional class; Figure S4: Forest plots of Cox proportional hazards models evaluating the association between the structural risk score and cardiovascular mortality. Hazard ratios (HRs) and 95% confidence intervals (CIs) are shown for each covariate included in the models. (A) The unadjusted model. (B) A model adjusted for age and sex. (C) An extended sensitivity model additionally adjusted for diabetes, arrhythmia, systolic blood pressure, resting heart rate, and major cardiovascular medications. Abbreviations: ACEI, angiotensin-converting enzyme inhibitor; ARB, angiotensin receptor blocker; LDL, low-density lipoprotein cholesterol; BP, blood pressure; CKD, chronic kidney disease; NYHA, New York Heart Association functional class; Figure S5: Hierarchical clustering dendrogram (Ward linkage). Dendrogram of hierarchical clustering using Ward’s minimum variance method applied to echocardiographic variables. Cutting at k = 3 yielded a broadly similar structure to PAM clustering, with the major low-risk cluster remaining stable across methods; Table S1: Mean and SD of Structural Variables; Table S2: Missing Data and Multiple Imputation Procedures; Table S3: Full Version of Baseline Characteristics by Risk Group; Table S4: Principal Component Loadings of Structural Echocardiographic Variables; Table S5: Cross tabulation comparing PAM clustering (k = 3) with hierarchical clustering using Ward linkage (k = 3).
