# Supplementary material for: Multidimensional Structural Echocardiographic Patterns and Risk Score for Prognostic Stratification in Ischemic Cardiomyopathy
Source: J Clin Med. 2026 Jun 5;15(11):4386. doi: 10.3390/jcm15114386 (PMC13257503; doi:10.3390/jcm15114386)
Supplement: Supplementary file 1 [file jcm-15-04386-s001.zip › Supplementary Table S1.pdf]

Supplementary Table S1. Mean and SD of Structural Variables

Variable	Mean	SD
LAd(mm)	43.516	5.365
LVEDD(mm)	58.562	6.967
LVESD(mm)	45.476	7.327
LVEF(%)	44.155	8.061
PAP(mmHg)	41.145	11.228
MR	2.596	0.945
IVS(mm)	9.303	1.447
PWT(mm)	8.810	1.109

Mean and standard deviation values for each echocardiographic variable in the derivation cohort.
