# Supplementary material for: Multidimensional Structural Echocardiographic Patterns and Risk Score for Prognostic Stratification in Ischemic Cardiomyopathy
Source: J Clin Med. 2026 Jun 5;15(11):4386. doi: 10.3390/jcm15114386 (PMC13257503; doi:10.3390/jcm15114386)
Supplement: Supplementary file 1 [file jcm-15-04386-s001.zip › Supplementary Table S2.pdf]

Supplementary Table S2. Missing Data and Multiple Imputation Procedures

Variable	Missing (n)	Missing (%)	Method	Predictors
Age	2	0.2	pmm	all other variables
Sex	1	0.1	logreg	age, BMI, comorbidities
BMI	180	18.2	pmm	age, sex, SBP, DBP
Smoking	17	1.72	logreg	age, sex, BMI, comorbidities
Drinking	15	1.52	logreg	age, sex, BMI, comorbidities
Family history	50	5.06	logreg	age, sex, comorbidities
Hypertension	0	0	—	—
Diabetes	0	0	—	—
Dyslipidemia	0	0	—	—
CKD	0	0	—	—
COPD	0	0	—	—
Asthma	0	0	—	—
AF	0	0	—	—
Arrhythmias	0	0	—	—
Depression	0	0	—	—
Thyroid dysfunction	0	0	—	—
Cancer	1	0.1	logreg	age, sex, comorbidities
SBP	17	1.72	pmm	age, sex, BMI, DBP, HR
DBP	16	1.62	pmm	age, sex, BMI, SBP, HR
Rest HR	16	1.62	pmm	age, sex, SBP, DBP

Variable	Missing (n)	Missing (%)	Method	Predictors
TG	34	3.44	pmm	age, sex, BMI, other lipids
TC	35	3.54	pmm	age, sex, BMI, other lipids
HDL	35	3.54	pmm	age, sex, BMI, other lipids
LDL	35	3.54	pmm	age, sex, BMI, other lipids
ApoA	36	3.64	pmm	age, sex, BMI, other lipids
ApoB	36	3.64	pmm	age, sex, BMI, other lipids
Lp(a)	37	3.74	pmm	age, sex, BMI, other lipids
HbA1c	63	6.37	pmm	age, sex, BMI, other lipids
NYHA	0	0	—	—
LAd	0	0	—	—
LVEDD	0	0	—	—
LVESD	0	0	—	—
LVEF	0	0	—	—
PAP	0	0	—	—
MR	0	0	—	—
IVS	0	0	—	—
PWT	0	0	—	—
ACEI	0	0	—	—
ARB	0	0	—	—
BB	0	0	—	—
Spironolactone	0	0	—	—

Variable	Missing (n)	Missing (%)	Method	Predictors
Loop diuretics	0	0	—	—
Thiazide diuretics	0	0	—	—
Digoxin	0	0	—	—
Aspirin	0	0	—	—
Clopidogrel	0	0	—	—
Nitrates	0	0	—	—
Statins	0	0	—	—
CCB	0	0	—	—

This table summarizes the missingness profile and imputation method for all variables included in the multiple imputation procedure. The outcome variable was not included in any imputation model. Comorbidities included hypertension, diabetes, CKD, COPD, AF, arrhythmias, thyroid dysfunction, and cancer. Lipids included TG, TC, HDL, LDL, ApoA, ApoB, Lp(a), and HbA1c.

Abbreviations: BMI, body mass index; CKD, chronic kidney disease; COPD, chronic obstructive pulmonary disease; AF, atrial fibrillation; SBP, systolic blood pressure; DBP, diastolic blood pressure; HR, heart rate; TG, triglycerides; TC, total cholesterol; HDL, high-density lipoprotein; LDL, low-density lipoprotein; ApoA, apolipoprotein A; ApoB, apolipoprotein B; Lp(a), lipoprotein(a); HbA1c, glycated hemoglobin; NYHA, New York Heart Association functional class; LAd, left atrial diameter; LVEDD, left ventricular end-diastolic diameter; LVESD, left ventricular end-systolic diameter; LVEF, left ventricular ejection fraction; PAP, pulmonary artery pressure; MR, mitral regurgitation; IVS, interventricular septal thickness; PWT, posterior wall thickness; ACEI, angiotensin-converting enzyme inhibitor; ARB, angiotensin receptor blocker; BB, beta-blocker; CCB, calcium channel blocker.
