# Supplementary material for: Multidimensional Structural Echocardiographic Patterns and Risk Score for Prognostic Stratification in Ischemic Cardiomyopathy
Source: J Clin Med. 2026 Jun 5;15(11):4386. doi: 10.3390/jcm15114386 (PMC13257503; doi:10.3390/jcm15114386)
Supplement: Supplementary file 1 [file jcm-15-04386-s001.zip › Supplementary Table S3.pdf]

Supplementary Table S3. Full Version of Baseline Characteristics by Risk Group.

Variable	Low	Medium	High	p
Age	67.32 ± 11.15	66.24 ± 10.03	64.98 ± 10.10	0.023
Sex				<0.001
Female	94 (26.0%)	35 (10.5%)	43 (14.7%)	
Male	268 (74.0%)	299 (89.5%)	250 (85.3%)	
BMI	23.72 ± 3.34	25.15 ± 8.08	23.90 ± 3.42	<0.001
Smoking				0.003
Current	93 (25.7%)	125 (37.4%)	93 (31.7%)	
Never	226 (62.4%)	162 (48.5%)	154 (52.6%)	
Used to	43 (11.9%)	47 (14.1%)	46 (15.7%)	
Drinking				0.039
Current	46 (12.7%)	66 (19.8%)	52 (17.7%)	
Never	306 (84.5%)	258 (77.2%)	226 (77.1%)	
Used to	10 (2.8%)	10 (3.0%)	15 (5.1%)	
Family history				0.190
No	309 (85.4%)	292 (87.4%)	241 (82.3%)	
Yes	53 (14.6%)	42 (12.6%)	52 (17.7%)	
Hypertension				<0.001
No	125 (34.5%)	79 (23.7%)	126 (43.0%)	
Yes	237 (65.5%)	255 (76.3%)	167 (57.0%)	
Diabetes				0.111
No	220 (60.8%)	180 (53.9%)	158 (53.9%)	
Yes	142 (39.2%)	154 (46.1%)	135 (46.1%)	
Dyslipidemia				0.002
No	239 (66.0%)	226 (67.7%)	229 (78.2%)	
Yes	123 (34.0%)	108 (32.3%)	64 (21.8%)	
CKD				0.004
No	334 (92.3%)	283 (84.7%)	251 (85.7%)	
Yes	28 (7.7%)	51 (15.3%)	42 (14.3%)	
COPD				0.565
No	333 (92.0%)	300 (89.8%)	264 (90.1%)	
Yes	29 (8.0%)	34 (10.2%)	29 (9.9%)	
Asthma				0.276
No	356 (98.3%)	330 (98.8%)	292 (99.7%)	
Yes	6 (1.7%)	4 (1.2%)	1 (0.3%)	
AF				0.101
No	324 (89.5%)	298 (89.2%)	245 (83.6%)	
Paroxysmal	26 (7.2%)	19 (5.7%)	32 (10.9%)	
Permanent	0 (0.0%)	2 (0.6%)	1 (0.3%)	
Persistent	12 (3.3%)	15 (4.5%)	15 (5.1%)	
Arrhythmias				0.011
No	309 (85.4%)	280 (83.8%)	225 (76.8%)	
Yes	53 (14.6%)	54 (16.2%)	68 (23.2%)	

Depression				0.879
No	359 (99.2%)	332 (99.4%)	292 (99.7%)	
Yes	3 (0.8%)	2 (0.6%)	1 (0.3%)	
Thyroid dysfunction				0.390
No	355 (98.1%)	329 (98.5%)	291 (99.3%)	
Yes	7 (1.9%)	5 (1.5%)	2 (0.7%)	
Cancer				0.484
No	347 (95.9%)	319 (95.5%)	285 (97.3%)	
Yes	15 (4.1%)	15 (4.5%)	8 (2.7%)	
SBP	125.52 ± 21.09	131.50 ± 22.24	124.60 ± 22.48	<0.001
DBP	72.95 ± 12.98	73.75 ± 13.30	73.03 ± 15.13	0.355
Rest HR	80.17 ± 15.12	78.97 ± 13.82	82.33 ± 14.53	0.006
TG	1.58 ± 1.10	1.76 ± 1.53	1.62 ± 1.51	0.010
TC	4.09 ± 1.13	3.97 ± 1.12	3.88 ± 1.06	0.058
HDL	1.04 ± 0.27	0.97 ± 0.25	0.97 ± 0.28	<0.001
LDL	2.57 ± 1.15	2.61 ± 1.30	2.58 ± 1.25	0.857
ApoA	1.14 ± 0.21	1.12 ± 0.21	1.10 ± 0.23	0.062
ApoB	0.85 ± 0.28	0.87 ± 0.37	0.84 ± 0.30	0.618
Lp(a)	0.30 ± 0.32	0.37 ± 0.58	0.38 ± 0.55	0.548
Hba1c	7.01 ± 1.93	7.03 ± 1.81	7.11 ± 1.84	0.119
NYHA				<0.001
II	194 (53.6%)	143 (42.8%)	70 (23.9%)	
III	148 (40.9%)	172 (51.5%)	184 (62.8%)	
IV	20 (5.5%)	19 (5.7%)	39 (13.3%)	
LAd	39.81 ± 3.93	43.63 ± 3.93	47.96 ± 4.90	<0.001
LVEDD	52.24 ± 3.85	60.34 ± 3.90	64.35 ± 6.46	<0.001
LVESD	38.72 ± 3.47	46.73 ± 3.77	52.40 ± 6.63	<0.001
LVEF	49.94 ± 5.53	44.10 ± 5.92	37.07 ± 7.10	<0.001
PAP	37.82 ± 8.42	37.16 ± 8.42	49.79 ± 12.30	<0.001
MR	2.19 ± 0.66	2.20 ± 0.50	3.55 ± 0.95	<0.001
IVS	8.92 ± 1.14	9.99 ± 1.56	8.99 ± 1.37	<0.001
PWT	8.70 ± 0.85	9.13 ± 1.24	8.58 ± 1.16	<0.001
ACEI				0.082
No	213 (58.8%)	222 (66.5%)	191 (65.2%)	
Yes	149 (41.2%)	112 (33.5%)	102 (34.8%)	
ARB				0.089
No	276 (76.2%)	230 (68.9%)	210 (71.7%)	
Yes	86 (23.8%)	104 (31.1%)	83 (28.3%)	
BB				0.017
No	49 (13.5%)	39 (11.7%)	57 (19.5%)	
Yes	313 (86.5%)	295 (88.3%)	236 (80.5%)	
Spironolactone				<0.001
No	197 (54.4%)	121 (36.2%)	89 (30.4%)	
Yes	165 (45.6%)	213 (63.8%)	204 (69.6%)	

Loop diuretics				<0.001
No	201 (55.5%)	127 (38.0%)	99 (33.8%)	
Yes	161 (44.5%)	207 (62.0%)	194 (66.2%)	
Thiazide diuretics				0.201
No	328 (90.6%)	289 (86.5%)	263 (89.8%)	
Yes	34 (9.4%)	45 (13.5%)	30 (10.2%)	
Digoxin				0.499
No	349 (96.4%)	320 (95.8%)	277 (94.5%)	
Yes	13 (3.6%)	14 (4.2%)	16 (5.5%)	
Aspirin				0.378
No	51 (14.1%)	54 (16.2%)	53 (18.1%)	
Yes	311 (85.9%)	280 (83.8%)	240 (81.9%)	
Clopidogrel				0.235
No	137 (37.8%)	145 (43.4%)	127 (43.3%)	
Yes	225 (62.2%)	189 (56.6%)	166 (56.7%)	
Nitrates				<0.001
No	165 (45.6%)	99 (29.6%)	104 (35.5%)	
Yes	197 (54.4%)	235 (70.4%)	189 (64.5%)	
Statins				0.014
No	31 (8.6%)	28 (8.4%)	43 (14.7%)	
Yes	331 (91.4%)	306 (91.6%)	250 (85.3%)	
CCB				0.002
No	330 (91.2%)	292 (87.4%)	280 (95.6%)	
Yes	32 (8.8%)	42 (12.6%)	13 (4.4%)	

This table summarizes demographic, clinical, laboratory, and echocardiographic characteristics across the three preoperative structural risk groups. Continuous variables are presented as mean ± SD or median (IQR) and were compared across groups using ANOVA or Kruskal–Wallis tests as appropriate. Categorical variables were compared using Chi-square or Fisher's exact tests. Abbreviations: BMI, body mass index; CKD, chronic kidney disease; COPD, chronic obstructive pulmonary disease; AF, atrial fibrillation; SBP, systolic blood pressure; DBP, diastolic blood pressure; HR, heart rate; TG, triglycerides; TC, total cholesterol; HDL, high-density lipoprotein; LDL, low-density lipoprotein; ApoA, apolipoprotein A; ApoB, apolipoprotein B; Lp(a), lipoprotein(a); HbA1c, glycated hemoglobin; NYHA, New York Heart Association functional class; LAd, left atrial diameter; LVEDD, left ventricular end-diastolic diameter; LVESD, left ventricular end-systolic diameter; LVEF, left ventricular ejection fraction; PAP, pulmonary artery pressure; MR, mitral regurgitation; IVS, interventricular septal thickness; PWT, posterior wall thickness; ACEI, angiotensin-converting enzyme inhibitor; ARB, angiotensin receptor blocker; BB, beta-blocker; CCB, calcium channel blocker.
