# Supplementary material for: Multidimensional Structural Echocardiographic Patterns and Risk Score for Prognostic Stratification in Ischemic Cardiomyopathy
Source: J Clin Med. 2026 Jun 5;15(11):4386. doi: 10.3390/jcm15114386 (PMC13257503; doi:10.3390/jcm15114386)
Supplement: Supplementary file 1 [file jcm-15-04386-s001.zip › Supplementary Table S4.pdf]

Supplementary Table S4. Principal Component Loadings of Structural Echocardiographic Variables

Variable	PC1	PC2	PC3
LAd	0.78	0.18	0.15
LVEDD	0.87	0.14	-0.28
LVESD	0.93	0.06	-0.32
LVEF	-0.76	0.12	0.30
PAP	0.51	-0.15	0.65
MR	0.58	-0.17	0.58
IVS	-0.01	0.86	0.13
PWT	-0.06	0.85	0.10

Loadings represent the correlation between each echocardiographic variable and the corresponding principal component. PC1 reflects the dominant axis of cardiac remodeling and volume overload, primarily driven by chamber size, systolic function, pulmonary pressures, and mitral regurgitation. PC2 captures ventricular wall thickness characteristics. PC3 explains a smaller proportion of variance.

Abbreviations: LAd, left atrial diameter; LVEDD, left ventricular end-diastolic diameter; LVESD, left ventricular end-systolic diameter; LVEF, left ventricular ejection fraction; PAP, pulmonary artery pressure; MR, mitral regurgitation; IVS, interventricular septal thickness; PWT, posterior wall thickness.
