# Supplementary material for: Multidimensional Structural Echocardiographic Patterns and Risk Score for Prognostic Stratification in Ischemic Cardiomyopathy
Source: J Clin Med. 2026 Jun 5;15(11):4386. doi: 10.3390/jcm15114386 (PMC13257503; doi:10.3390/jcm15114386)
Supplement: Supplementary file 1 [file jcm-15-04386-s001.zip › Supplementary Table S5.pdf]

Supplementary Table S5. Cross-tabulation of PAM vs. Hierarchical clustering (k = 3)

PAM cluster	HC cluster 1	HC cluster 2	HC cluster 3
1	46	262	26
2	0	362	0
3	26	104	163

Cross-tabulation comparing PAM clustering (k = 3) with hierarchical clustering using Ward linkage (k = 3).
