# Supplementary figures and images for: Multidimensional Structural Echocardiographic Patterns and Risk Score for Prognostic Stratification in Ischemic Cardiomyopathy
Source: J Clin Med. 2026 Jun 5;15(11):4386. doi: 10.3390/jcm15114386 (PMC13257503; doi:10.3390/jcm15114386)

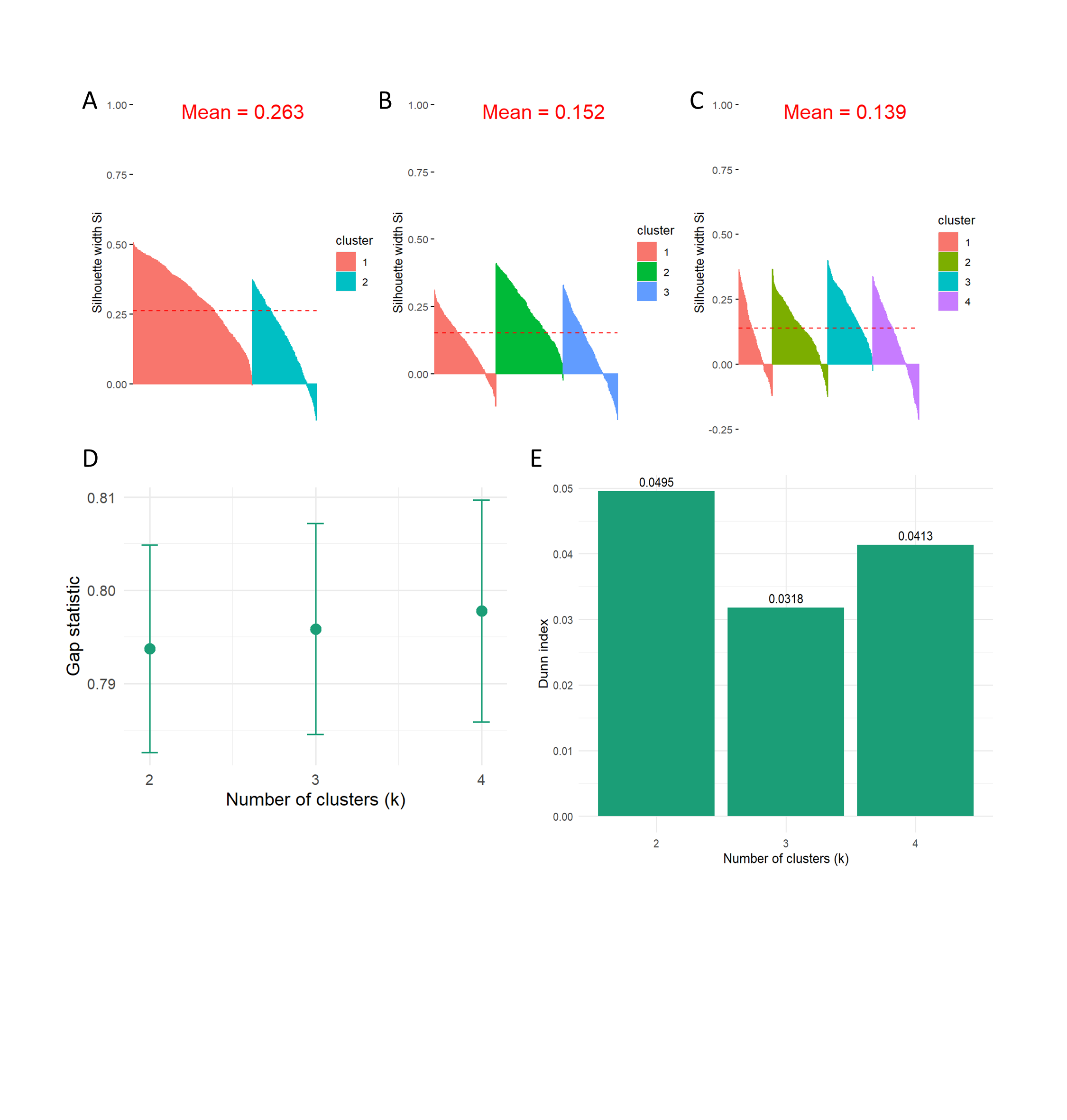

Supplement: Supplementary file 1 [file jcm-15-04386-s001.zip › Supplementary Figure S1.png]

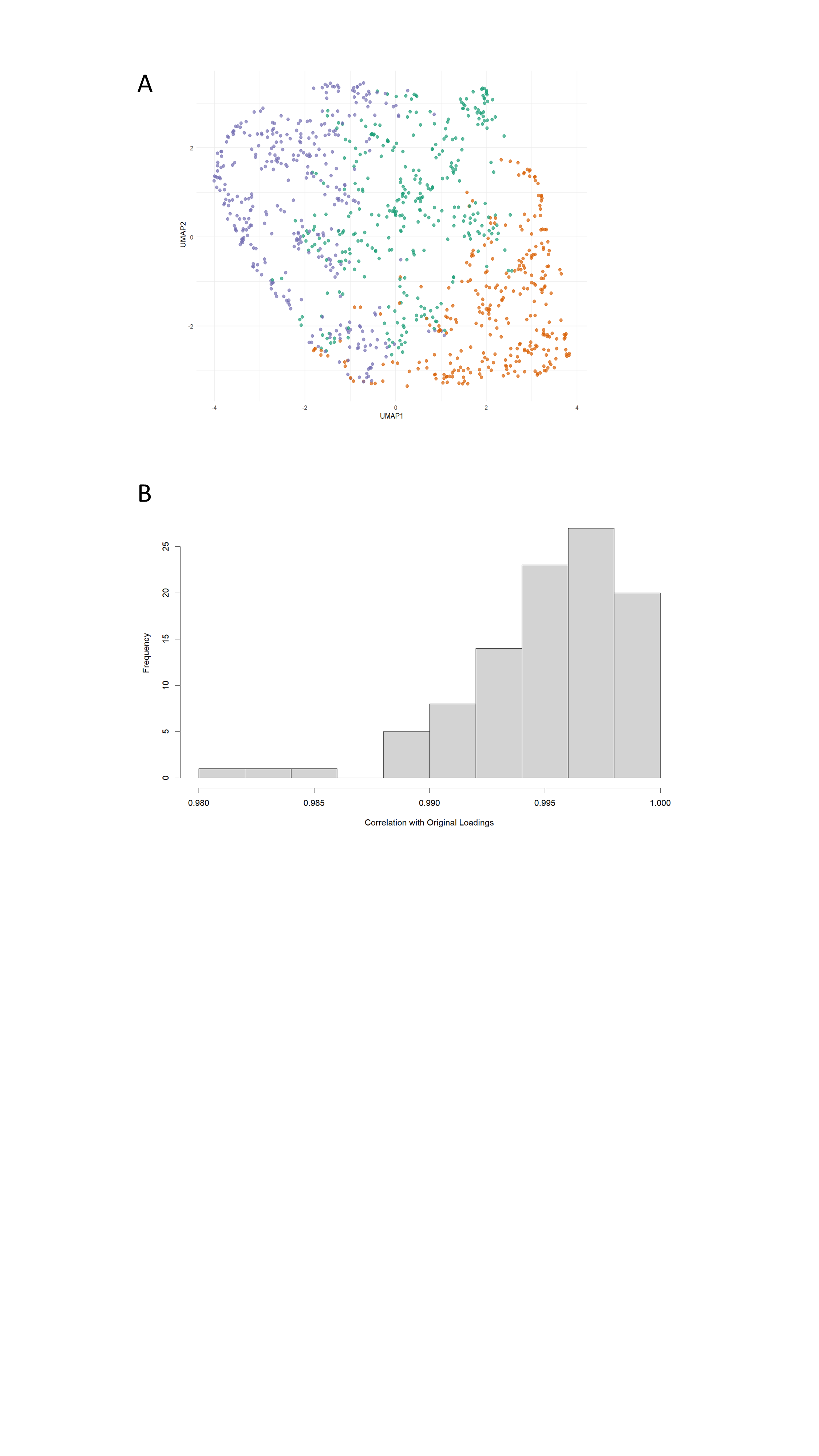

Supplement: Supplementary file 1 [file jcm-15-04386-s001.zip › Supplementary Figure S2.png]

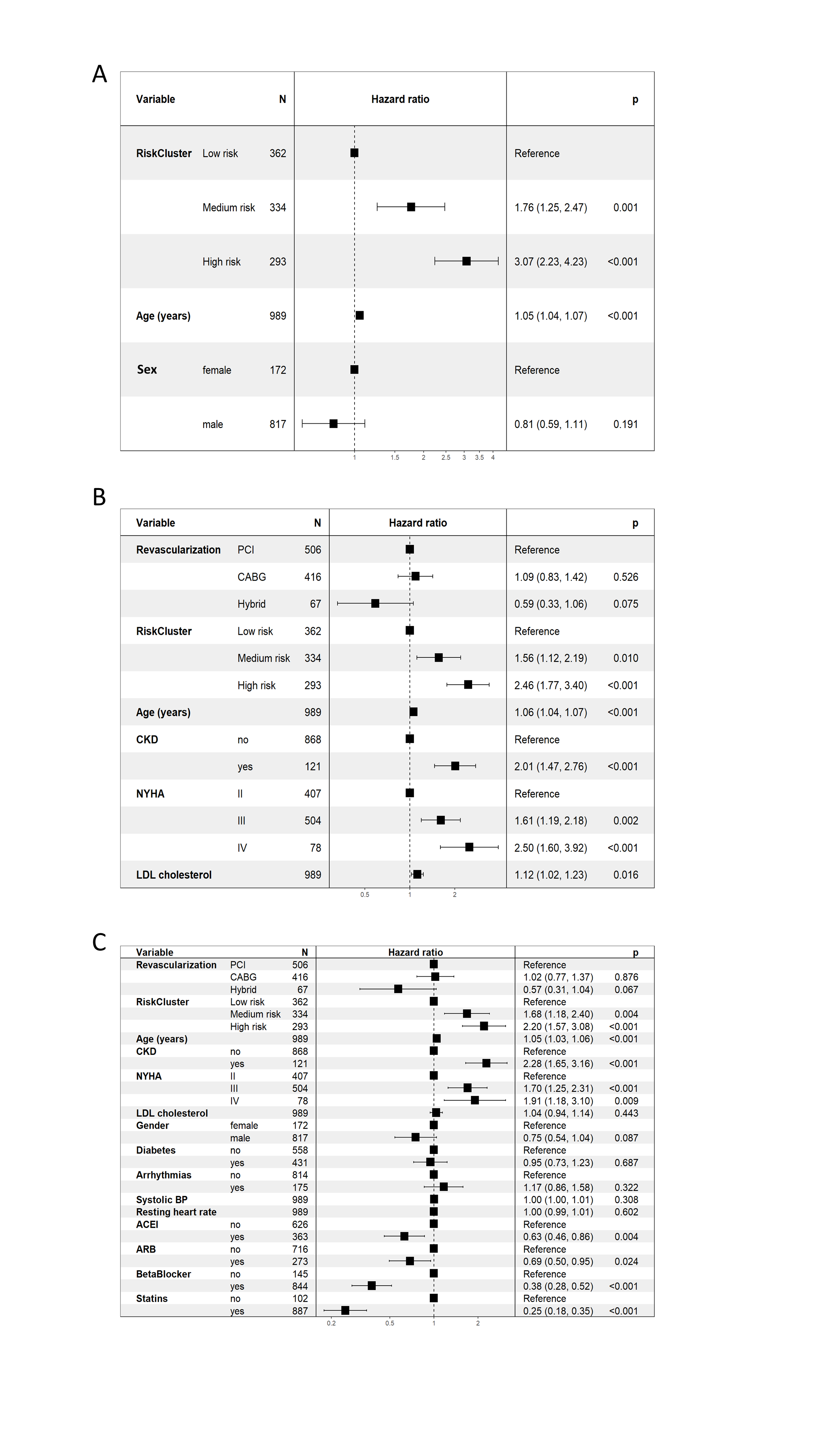

Supplement: Supplementary file 1 [file jcm-15-04386-s001.zip › Supplementary Figure S3.png]

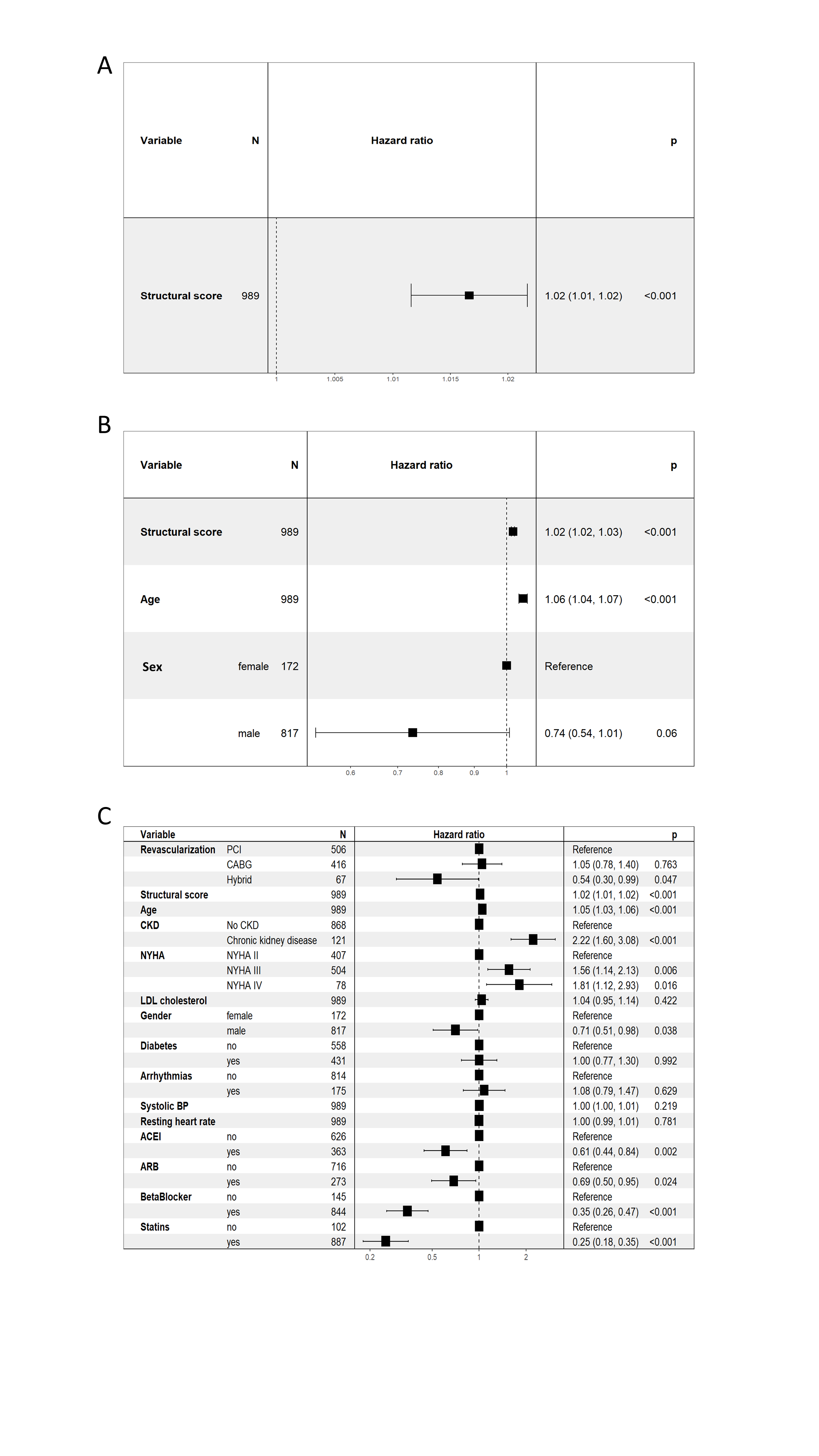

Supplement: Supplementary file 1 [file jcm-15-04386-s001.zip › Supplementary Figure S4.png]

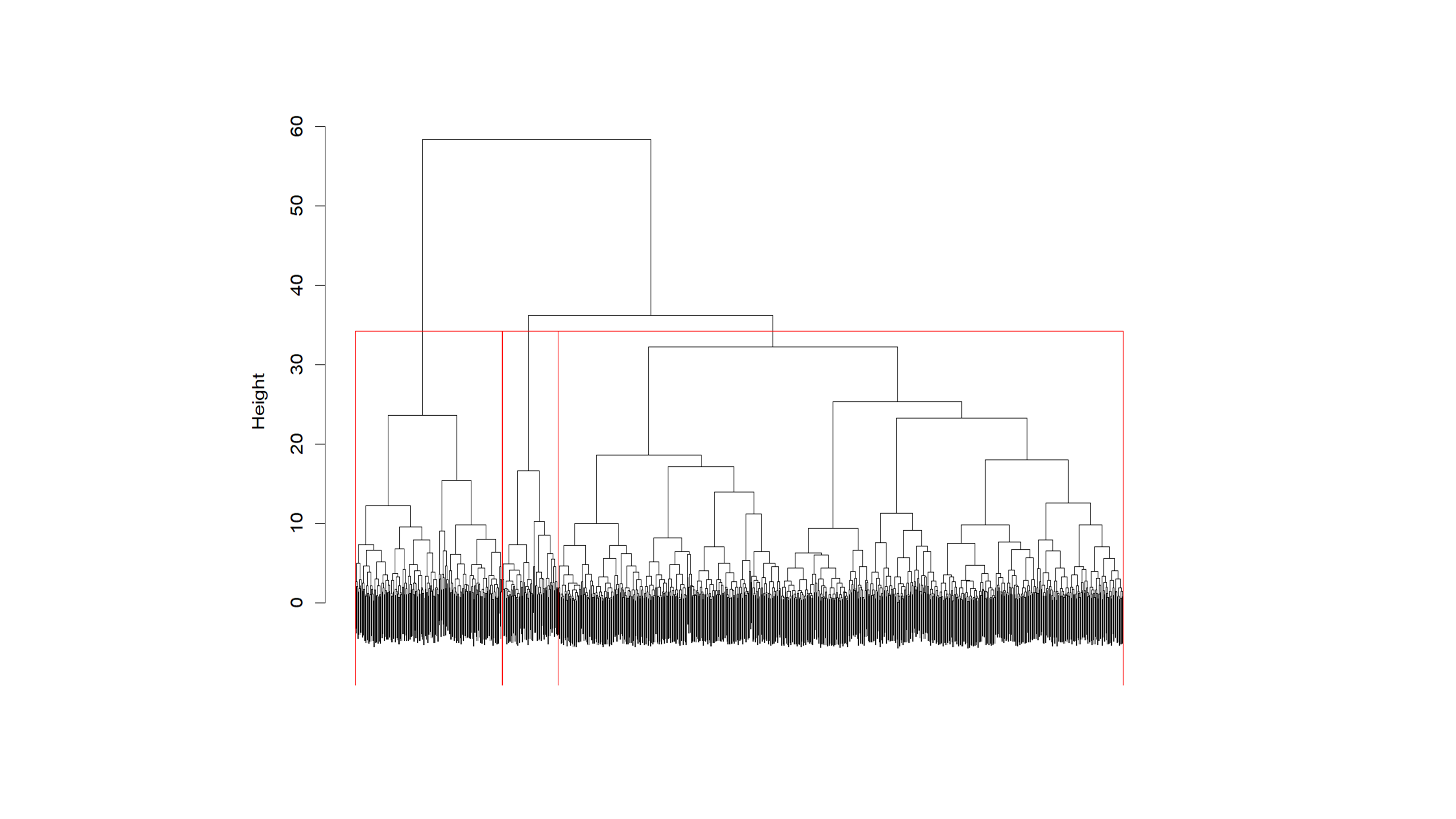

Supplement: Supplementary file 1 [file jcm-15-04386-s001.zip › Supplementary Figure S5.png]
